# Reprogramming of connexin landscape fosters fast gap junction intercellular communication in human papillomavirus-infected epithelia

**DOI:** 10.3389/fcimb.2023.1138232

**Published:** 2023-05-16

**Authors:** Carmen Gallego, Agnieszka Jaracz-Ros, Marta Laganà, Françoise Mercier-Nomé, Séverine Domenichini, Amos Fumagalli, Philippe Roingeard, Michael Herfs, Guillaume Pidoux, Françoise Bachelerie, Géraldine Schlecht-Louf

**Affiliations:** ^1^ Inflammation, Microbiome and Immunosurveillance, INSERM UMR-996, Université Paris-Saclay, Orsay, France; ^2^ US31-UMS3679-Plateforme PHIC, Ingénierie et Plateformes au Service de l’Innovation Thérapeutique (IPSIT), INSERM, CNRS, Université Paris-Saclay, Orsay, France; ^3^ UMS-IPSIT Plateforme MIPSIT, Université Paris-Saclay, CNRS, Inserm, Ingénierie et Plateformes au Service de l’Innovation Thérapeutique, Orsay, France; ^4^ CNRS, UMR-5203, Institut de Génomique Fonctionnelle, Montpellier, France; ^5^ INSERM U1259, Université de Tours et CHRU de Tours & Plateforme IBiSA des Microscopies, PPF ASB, CHRU de Tours, Tours, France; ^6^ Laboratory of Experimental Pathology, GIGA-Cancer, University of Liège, Liège, Belgium; ^7^ INSERM UMR-S 1180, Université Paris-Saclay, Orsay, France

**Keywords:** human papillomavirus (HPV), viral replication, connexin, gap junction intercellular communication (GJIC), 3D epithelial cell culture, fluorescence loss in photobleaching (FLIP)

## Abstract

Human papillomaviruses (HPVs) are highly prevalent commensal viruses that require epithelial stratification to complete their replicative cycle. While HPV infections are most often asymptomatic, certain HPV types can cause lesions, that are usually benign. In rare cases, these infections may progress to non-replicative viral cycles associated with high HPV oncogene expression promoting cell transformation, and eventually cancer when not cleared by host responses. While the consequences of HPV-induced transformation on keratinocytes have been extensively explored, the impact of viral replication on epithelial homeostasis remains largely unexplored. Gap junction intercellular communication (GJIC) is critical for stratified epithelium integrity and function. This process is ensured by a family of proteins named connexins (Cxs), including 8 isoforms that are expressed in stratified squamous epithelia. GJIC was reported to be impaired in HPV-transformed cells, which was attributed to the decreased expression of the Cx43 isoform. However, it remains unknown whether and how HPV replication might impact on the expression of Cx isoforms and GJIC in stratified squamous epithelia. To address this question, we have used 3D-epithelial cell cultures (3D-EpCs), the only model supporting the productive HPV life cycle. We report a transcriptional downregulation of most epithelial Cx isoforms except Cx45 in HPV-replicating epithelia. At the protein level, HPV replication results in a reduction of Cx43 expression while that of Cx45 increases and displays a topological shift toward the cell membrane. To quantify GJIC, we pioneered quantitative gap-fluorescence loss in photobleaching (FLIP) assay in 3D-EpCs, which allowed us to show that the reprogramming of Cx landscape in response to HPV replication translates into accelerated GJIC in living epithelia. Supporting the pathophysiological relevance of our observations, the HPV-associated Cx43 and Cx45 expression pattern was confirmed in human cervical biopsies harboring HPV. In conclusion, the reprogramming of Cx expression and distribution in HPV-replicating epithelia fosters accelerated GJIC, which may participate in epithelial homeostasis and host immunosurveillance.

## 1 Introduction

Human Papillomaviruses (HPVs) are epitheliotropic non-enveloped double-stranded DNA viruses that originally got attention due to their causal role in cervical cancers (de Martel et al., 2017; Alizon et al., 2019; McBride, 2022). Indeed, albeit most HPV infections are controlled by the host responses, persistent infection with some HPV types (*e.g.* HPV16 or 18), referred to as high-risk (hr)HPVs, can induce pre-cancerous lesions. The latter may progress to higher-grade lesions, that do not produce viral particles but express high levels of HPV oncogenes, and eventually to cancer. Thus, despite an overall low virulence, the high prevalence of HPV infections results in a substantial disease burden, with hrHPVs causing 5% of human cancers (Schiffman et al., 2016). In addition to their established role in oncogenesis, HPVs are now recognized as members of the human microbiota, with more than 400 prevalent types identified in the skin and mucosa stratified squamous epithelia. Most HPVs are primarily associated with asymptomatic infections, some of them being acquired in early childhood (Foulongne et al., 2012; Pastrana et al., 2018; Liang and Bushman, 2021). However, despite HPVs being the most frequent eukaryotic viruses in the skin and vagina (Ma et al., 2014; Wylie et al., 2014), the impact of their replication on epithelial homeostasis remains largely unexplored. This limited knowledge can partly be explained by the fact that HPVs depend on epithelial stratification to complete their productive life cycle, which imposes the use of three-dimensional epithelial cell cultures (3D-EpCs) for studying HPV-host interplay in a pathophysiology-relevant setting (Meyers et al., 1992; Doorbar, 2016). Accordingly, the interactions of HPVs with epithelia have been mostly studied in terms of their carcinogenic role and have largely been inferred from studying human biopsies or transformed human cell lines.

Gap junction intercellular communication (GJIC) (Aasen et al., 2017; Laird and Lampe, 2022), which warrants the functional coupling of cells in stratified epithelia and supports their barrier function toward external threats, is one of the processes that has been reported to be impacted by HPVs’ oncogene expression, directly or as a potential consequence of their carcinogenetic action on keratinocytes (Oelze et al., 1995; Aasen et al., 2005; Sun et al., 2015; Klymenko et al., 2017). GJIC is ensured by connexins (Cxs), a family of 21 proteins including 8 members that are expressed in stratified squamous epithelia (Martin et al., 2014) with a specific pattern according to cell layer (Di et al., 2001; Laird and Lampe, 2018; Garcia-Vega et al., 2021). Cxs assemble in homo-or heteromeric structures called connexons. Connexons from adjacent cells can further dock to each other to form intercellular conduits, called gap junction (GJ) channels, allowing for exchanges of small-size molecules (<1 kDa) such as ions, metabolites, and second messengers between connected cells. GJ channels further aggregate into highly organized plaques that support these exchanges (Aasen et al., 2017).

The hypothesis that GJIC is impaired in the context of HPV-associated carcinogenesis stemmed from the seminal works of McNutt and Weinstein, who reported a reduction in GJ numbers in cervical carcinomas (McNutt and Weinstein, 1969; McNutt et al., 1971). Later, the pattern of Cx expression was reported to be altered in HPV-induced cervical intraepithelial neoplasia (CIN) in relationship with the severity of the grade (1/2/3) (Aasen et al., 2005). Consistent with a direct contribution of HPV in this modulation, the expression of the hrHPV E6 and E5 oncoproteins was shown to reduce the expression and modify the cellular distribution and phosphorylation pattern of Cx43 (Ennaji et al., 1995; Oelze et al., 1995; Tomakidi et al., 2000; Sun et al., 2015), the most broadly expressed Cx isoform (Aasen et al., 2017). Some of these studies have further associated Cx43 downregulation with GJIC impairment in E6- and E5-expressing cells using semiquantitative methods (Ennaji et al., 1995; Oelze et al., 1995), an association later proposed to feature epithelial dysplasia (King et al., 2000; Aasen et al., 2003). Altogether, these studies conveyed that, on the one hand, HPV-induced cell transformation is associated with reduced Cx43 expression and, on the other hand, Cx43 downregulation is sufficient to cause GJIC impairment. However, the genuine impact of HPV replication on the expression of the whole set of epithelial Cxs and on GJIC in a living stratified epithelium remains unknown, a question of critical importance considering the commensal carriage of HPVs in our epithelia.

To tackle this issue, we have used 3D-EpCs, the sole cell culture model supporting the complete HPV replicative cycle up to viral particle production (Meyers et al., 1992; Doorbar, 2016). Besides, 3D-EpCs provide a relevant system to study the Cx expression pattern and its relationship with GJIC (McLachlan et al., 2006; Eng et al., 2016). First, by analyzing the impact of HPV18 replication on Cx isoforms expression in 3D-EpCs, we evidence a general transcriptional reprogramming of the main epithelial Cx isoforms detected in these tissues. We further show that the protein levels of the most down- and up-regulated Cx isoforms, Cx43 and Cx45, respectively, are modified accordingly and that their subcellular and tissue distribution is reprogrammed. Second, by implementing in 3D-EpCs the sensitive and non-invasive Fluorescence Loss in Photobleaching (FLIP) microscopy approach to quantify GJIC (Dukic et al., 2018), we demonstrate that this dynamic process is accelerated rather than blunted in tissues supporting HPV replication, as a likely consequence of Cx reprogramming. Finally, by analyzing HPV18-positive pre-cancerous lesions, known to replicate the virus (Lowy and Schiller, 2006; Schiffman et al., 2016), we validate the relevance of the pattern of Cx43 and Cx45 reprogramming in terms of human pathophysiology.

## 2 Materials and methods

### 2.1 Ethics statement

Cervical tissue specimens were retrieved from the Biobank of the University hospital center of Liege (Belgium) with the approval of the local ethics committee (CHU-ULiege). All samples were selected from an already-existing collection and were analyzed anonymously. Both the Biobank and the researchers strictly follow the rules imposed by current Belgian legislation. All samples contained areas of normal squamous epithelium (ectocervix/transformation zone) and HPV18-positive lesions, validated by experienced pathologists as well as the detection of HPV18 (Abbott RealTime High Risk HPV test, Abbott, Chicago, IL, USA).

### 2.2 Cell lines

NIKS (nearly-diploid immortalized keratinocytes), primary-like keratinocytes that keep normal growth and differentiation features line (Allen-Hoffmann et al., 2000), were maintained sub-confluent on mitomycin C-treated 3T3 (3T3 MMC) feeder cells (both cell lines kindly provided by Dr. Paul F. Lambert) as described earlier (Lambert et al., 2005). Briefly, NIKS were seeded on top of 3T3 MMC in F incomplete (FI) medium (F12:DMEM (3:1) (Gibco, Life technologies), 5% FCS, 1% penicillin/streptomycin (Gibco, Life technologies), 0.4 µg/mL hydrocortisone (cat#386698, Calbiochem, EMD Millipore), 24.2 µg/mL adenine (cat#A9795, Sigma-Aldrich), 5 µg/mL insulin (cat#I6634, Sigma-Aldrich) and 0.1 nM cholera toxin (cat#C8052, Sigma-Aldrich) and once they were attached, the medium was changed to F complete medium (FI with 0.1 ng/mL EGF (cat#E9644, Sigma-Aldrich). The medium was changed every other day. NHDF (normal human dermal fibroblasts, Lonza) were maintained in Ham’s Nutrient F12 mix media (Gibco, Life Technologies) supplemented with 10% FCS and 1% penicillin/streptomycin. Cells were kept at 37°C, 5% CO_2_ and regularly checked for mycoplasma contamination using MycoAlert™ Mycoplasma Detection Kit (cat#LT07-218, Lonza).

### 2.3 Generation of HPV-expressing NIKS cells

Circular HPV18 genome was prepared as previously described (Lambert et al., 2005). NIKS were plated (5.5 x 10^4^ cells/cm^2^) on blasticidin-resistant 3T3 MMC feeders and co-transfected the next day with 0.8 μg of circular HPV18 genome and 0.2 μg of blasticidin-resistance plasmid pcDNA6 per million of cells using Effectene Transfection Reagent (cat#301425, QIAGEN), according to manufacturer’s instructions. Blasticidin selection (7 μg/mL; cat#ant-bl-1, Invivogen) was performed for 6 days.

### 2.4 3D-EpCs

HPV18-positive and control (non-transfected) NIKS cells were used to generate 3D-EpCs following the previously described protocol (Lambert et al., 2005). Briefly, a dermal equivalent, made of rat-tail collagen type I (cat#08-115, Merck) and 0.75 x 10^6^/mL NHDF cells, was placed onto transwell inserts, and 1.5 x 10^6^ NIKS cells were seeded on top of it. Inserts were then submerged in deep wells containing keratinocyte plating-medium (FI with 0.5% FCS and 1.88 mM CaCl_2_) and after 4 days, cultures were lifted to expose them to the air-liquid interface. The medium was changed every other day (FI with 10 µM of C8:0 (1,2-dioctanoyl-sn-glycerol, cat#317505, Calbiochem EMD Millipore)), and cultures were harvested at day 15-18 post-seeding. 3D-EpCs were fixed in 4% formaldehyde and then embedded in paraffin before sectioning. Cell pellets from NIKS (day 0) and epidermal sheets from day 15-18 3D-EpCs were recovered and frozen at -80°C for further RNA and protein extraction.

### 2.5 Calcium-induced differentiation in 2D culture

NIKS cells were induced to differentiate in high-calcium (1.5 mM CaCl_2_) FI medium without FCS, as described in (Meuris et al., 2016). Briefly, cells were plated in the absence of 3T3 MMC in FI medium. 18h post-seeding, the medium was switched to the high-calcium medium, and cells were harvested at 0h, 48h, or 96h post-seeding. The medium was changed every day.

### 2.6 RNA extraction

Total RNA was extracted from cells or epidermal sheets using RNeasy Mini Plus kit or RNeasy Fibrous Tissue Mini kit (cat#74134 and cat#74704, QIAGEN), respectively, according to manufacturer’s instructions. RNA quantity and quality were assessed using NanoDrop™ One (Thermo Fisher Scientific).

### 2.7 Real time quantitative PCR

cDNA synthesis was done using M-MLV reverse transcription (cat#28023-013, Invitrogen) and quantitative PCR was performed using SybrGreen I or Probes Master 480 mix (cat#04707516001 and cat#04707494001, Roche) together with Taqman Gene Expression Assays (Applied biosystems, Thermo Fisher Scientific) or Universal ProbeLibrary probes (Roche, Sigma-Aldrich) in a LightCycler^®^ 480 Instrument II (Roche). Gene expression was normalized to GAPDH expression levels. Primers, probes, and assay IDs are listed in **Supplementary Table 1**.

### 2.8 Western blotting

Cell pellets and epidermal sheets were resuspended in lysis buffer (NP40 Cell Lysis Buffer cat #FNN0021, Thermo Fisher Scientific supplemented with Protease Inhibitor Cocktail Set I – Calbiochem cat# 539131, Sigma-Aldrich and Phosphatase Inhibitor Cocktail Set V, 50X cat# 524629, Millipore). Protein extracts were sonicated with Vibra-Cell™, CV17, BioBlock Scientific for 10 pulses at tune 50%, cycle 20%, power 5. Protein concentration was determined with the BCA Protein Assay kit (cat#23227, Pierce™ Thermo Fisher Scientific). Equal amounts of protein were loaded and resolved in SDS-polyacrylamide gel (Nupage 4%-12% Bis-Tris, cat#NP0321BOX or cat#NP0322BOX, Invitrogen, Thermo Fisher Scientific and Nupage 10% Bis-Tris, cat#NP0301BOX or cat#NP0303BOX, Invitrogen, Thermo Fisher Scientific) and transferred to PVDF membrane (Immobilon-P cat#IPVH00010, Merck Millipore). Membranes were incubated at 4°C overnight with the following primary antibodies: anti-Cx43 (1/10000, Sigma-Aldrich), anti-Cx45 (1/70, Santa Cruz Biotechnology) and anti-HPV18-E6 (1 µg/mL, Arbor Vita Corporation). Alpha-tubulin (1/2000, Sigma-Aldrich), GAPDH (1/2000, Thermo Fisher Scientific), and Cyclophilin B (1/1000, Thermo Fisher Scientific) were used as loading controls. Membranes were then incubated with the corresponding HRP-conjugated secondary antibodies (anti-mouse and anti-rabbit, cat#NA9340 and cat#NA9310, GE Healthcare). Protein detection was performed using Immobilon Western Chemiluminescent HRP kit (cat#WBKLS0500, Merck Millipore), and images were acquired and analyzed with UVP imaging system. Antibodies are listed in **Supplementary Table 2**.

### 2.9 Histological analysis

Paraffin-embedded tissue sections (5 µm thick) were deparaffinized and rehydrated. Tissue architecture was evaluated with Hematoxylin-Eosin (HE) coloration. Antigen retrieval was done in citrate buffer (pH 6.0), and sections were blocked with PBS 5% BSA or PBS 5% BSA 0.2% Triton X-100 (permeabilization buffer for nuclear antigens). Sections were incubated overnight at 4°C with the following primary antibodies diluted in PBS 5% BSA: anti-Cx43 (1/2000, Sigma-Aldrich), anti-phospho-Cx43 (Ser373) (1/100, Invitrogen, Thermo Fisher Scientific), anti-Cx45 (1/50, R&D Bio-techne), anti-HPV18-E4 (1/100, kindly provided by Dr. J. Doorbar), anti-HPV L1 (1/50, Dako), anti-Ki-67 (1/75, Abcam), anti-filaggrin (1/100, Abcam), anti-p16 INK4A (1/100, Santa Cruz Biotechnology) and anti-cornulin (1/500, Proteintech). Immunohistochemistry was performed for L1 detection using the Detection System Peroxidase/DAB+ kit (cat#K5001, Dako) and tissue was counterstained with Hematoxylin. For immunofluorescence, appropriate Alexa Fluor 594- or 488-conjugated secondary antibodies (1/200, anti-mouse and anti-rabbit, cat#A-11005, cat#A-11012, and Cat#A-11029, Invitrogen, Thermo Fisher Scientific) together with Hoechst 33342 to counterstain nuclei (1/500, cat#H3570, Life technologies, Thermo Fisher Scientific) were applied for 1h at room temperature. Sections were mounted with PermaFluor™ Aqueous Mounting Medium (cat#TA-030-FM, Thermo Fisher Scientific) and slides were scanned using the digital scanner NanoZoomer 2.0-RS (Hamamatsu) (**Supplementary Table 2**). Quantification of Cx fluorescence intensity was performed with the FIJI software (https://imagej.net/Fiji) (Schindelin et al., 2012; Schneider et al., 2012). We used the multi-point tool on red fluorescence channel in order to manually select and measure intensity of 30 to 50 different points for each region of interest (membrane or cytoplasm) and for each image (3-4 fields per 3D-EpC). Values were exported to Excel and Prism-GraphPad for statistical analyzes and graphical representations.

### 2.10 *In situ* hybridization

As previously detailed (Mirkovic et al., 2015; Herfs et al., 2018), carcinogenic HPV DNA was detected in paraffin-embedded 3D-EpCs cultures by *in situ* hybridization using the Ventana INFORM HPV III family 16 probes (Ventana Medical Systems, Tucson, AZ, USA). This kit allows the detection of 12 hr HPV genotypes (HPV16, 18, 31, 33, 35, 39, 45, 51, 52, 56, 58, and 66). The manufacturer’s recommendations were strictly followed, and, in the end, Red Counterstain II (Ventana Medical Systems) was used.

### 2.11 Electron microscopy

3D-EpCs were harvested at day 18 and fixed for 72h in 4% formaldehyde and 1% glutaraldehyde (Sigma-Aldrich, St. Louis, MO) in 0.1 M phosphate buffer (pH 7.2). Samples were washed with PBS and post-fixed by incubation with 2% osmium tetroxide (Agar Scientific, Stansted, UK) for 1h. 3D-EpCs were then fully dehydrated in a graded series of ethanol solutions (70%, 90% and 100%) and propylene oxide (100%). The samples were impregnated with a 1:1 mixture of propylene oxide/Epon resin (Sigma) and then incubated overnight in pure resin. They were then embedded in Epon resin and left to polymerize for 48h at 60°C. Ultrathin sections (80 nm) were cut with an EM UC7 ultramicrotome (Leica Microsystems, Wetzlar, Germany). Contrast staining was performed with 2% uranyl acetate (Agar Scientific) and 5% lead citrate (Sigma-Aldrich), and sections were then observed in a transmission electron microscope (JEOL 1400 Plus, Tokyo, Japan) equipped with a digital camera (Gatan OneView, Pleasanton, CA).

### 2.12 Immunocytofluorescence

NIKS cells were seeded in chambered microscope slides (Lab-Tek II, cat#154941, Nunc, Thermo Fisher Scientific). Cells were washed with PBS and fixed in 4% formaldehyde for 15 min at room temperature, then blocked with PBS 5% BSA for 1h at room temperature. Cells were incubated with anti-Cx43 antibody (1/1000, Sigma-Aldrich) for 2h at room temperature diluted in PBS 5% BSA. Like immunofluorescence in sections, Alexa Fluor 594-conjugated secondary antibody and Hoechst 33342 were applied for 1h at room temperature. Slides were mounted and scanned.

### 2.13 Fluorescence loss in photobleaching

To evaluate GJIC, FLIP microscopy technique was set up in 3D-EpCs. Epidermal sheets were recovered from 3D-EpCs and incubated dermal side floating down in µ-Dish 35 mm (cat#81156, ibidi) with 10 µM calcein Red-Orange AM (cat#C34851, Invitrogen, Thermo Fisher Scientific) for 1 h at room temperature in an orbital shaker. They were washed in PBS and any remaining liquid was removed by aspiration. Epidermal sheets were placed inside the temperature-controlled chamber (temperature and CO_2_) of the microscope and imaged for FLIP analysis according to previous protocols (Dukic et al., 2018) with the following modifications: images were acquired through a 40x 1.3NA objective and an area of ~22 µm^2^ was photobleached using a 561 nm laser delivering 2.5mW in the suprabasal layers. Images were acquired at every time point with an attenuated 561 nm laser.

### 2.14 FLIP analysis and quantification

Gap-FLIP images were acquired at every time point and processed with Metamorph software to generate time-lapses, which were then analyzed with ImageJ software. HPV epidermal sheet time-lapse was corrected for sample drifting using Motion 2D software. Mean grey intensity was measured for each cell type (described in **Figure 3**) during the first 280 s of bleaching. Background values were taken from areas where no cells were observed. Initial fluorescence was considered 100% after subtraction of background values and correction for autobleaching and fluorescence over time was calculated as the percentage of the initial fluorescence. One-phase decay curves adjustment was done in GraphPad Prism and half-life values and span (mobile fraction) were extracted from the analysis.

### 2.15 Statistical analysis

All statistical analyses were done in GraphPad Prism (version 7). Mann-Whitney test was used to compare samples and significance was established at p-value<0.05. *p-value<0.05, **p-value<0.01, ****p-value<0.001. The number of independent experiments and of replicates or samples are indicated in each legend.

## 3 Results

### 3.1 HPV replication induces transcriptional reprogramming of Cx expression in 3D-EpCs

**Figure 1:**
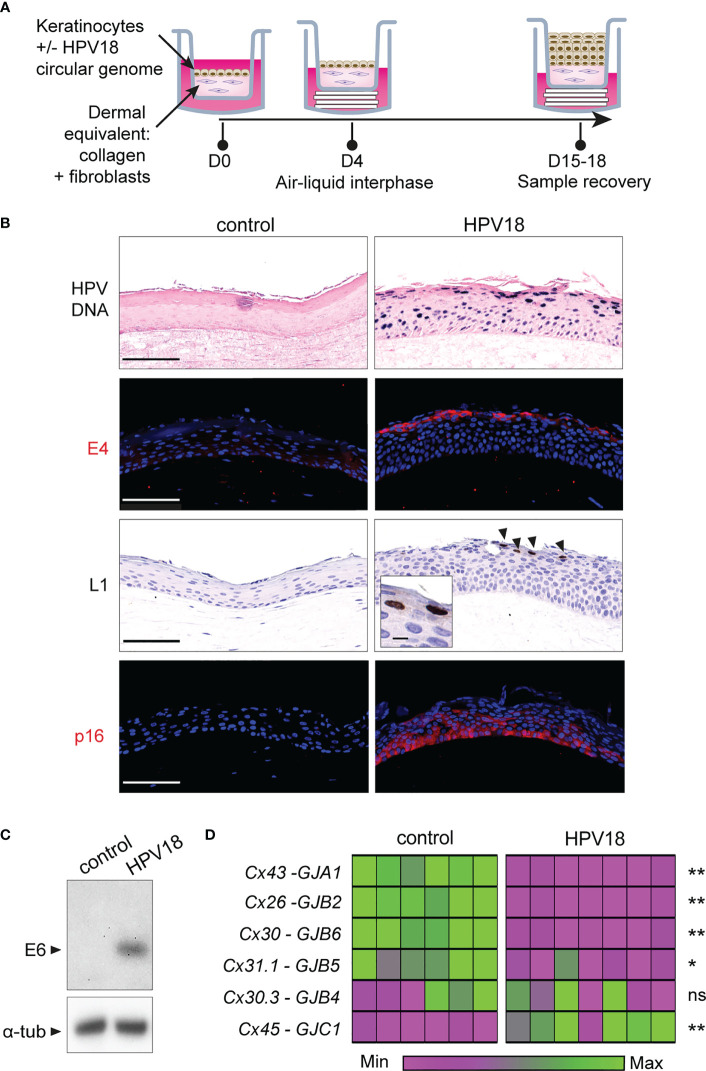
HPV-3D-EpCs show a transcriptomic reprogramming of Cx gene expression. **(A)** Schematic representation of the protocol for 3D-EpC generation. Keratinocytes, transfected or not with HPV18 circular genome, are seeded on the top of a dermal equivalent made of primary fibroblasts embedded in a collagen matrix, in a transwell insert. At day 4 post-seeding, insets are lifted using cotton pads to expose the tissue to the air-liquid interphase. The culture medium is changed every two days. At day 15-18, inserts are removed, and 3D-EpCs are collected for sample processing. **(B)** Representative images of control- and HPV-3D-EpCs showing, from top to bottom, *in situ* hybridization for HPV18 genome (HPV DNA) and staining of the replication-associated HPV18-E4 and HPV18-L1 proteins (arrowheads; Inset, magnification of L1-positive cells) as well as of p16^INK4a^ (p16). Hoechst was used for nuclei detection in immunostainings. Scale bar=100 µm, inset scale bar=10 µm. Control n=5-16, HPV18 n=5-13. Data are from 5 to 6 independent experiments. **(C)** Western blot of HPV18-E6 oncoprotein expression in control- and HPV-3D-EpCs. Representative of 5 independent experiments. α-tub, alpha-Tubulin **(D)** Heatmap showing the relative Cx isoform mRNA levels, measured by RT-qPCR in the epithelial sheets of 3D-EpCs after sample recovery at day 15-18 post-seeding. Each column represents an individual 3D-EpC, and each line corresponds to a Cx type (Protein/gene names are on the left). For each gene, normalized gene expression is displayed in the heatmap as a fold change to the mean values of the control samples from each experiment. The lower expression is mapped to magenta and the higher expression to green. The color gradient is independent for each Cx type. The statistical significance of the difference between control and HPV-3D-EpCs is shown on the right. *p < 0.05, **p < 0.01; ns, non-significant. Data are from 3 independent experiments with a total of 6 control- and 7 HPV-3D-EpCs.

The HPV replicative life cycle is tightly linked to the differentiation program of stratified epithelia, as the coordinated expression pattern of the eight epithelial Cxs along with cell layers also is (Di et al., 2001; Laird and Lampe, 2018; Garcia-Vega et al., 2021). Therefore, we generated 3D-EpCs with spontaneously immortalized NIKS keratinocytes (Allen-Hoffmann et al., 2000), transfected or not with circularized HPV18 genome (Lambert et al., 2005), to investigate the outcomes of HPV replication in terms of Cx isoform expression (**Figure 1A**).

As expected, control NIKS keratinocytes developed into a stratified epithelium within 15-18 days, with some dividing basal keratinocytes (Ki-67 staining), while those from the granular layers expressed the cornulin and filaggrin differentiation markers (**Figure S1**, left panels). The HPV18-expressing keratinocytes formed an epithelium with expanded basal and spinous layers as well as reduced cornification of the upper layers (**Figure S1**, right panels) (Flores et al., 1999; Lambert et al., 2005), in accordance with their exacerbated proliferation (Ki-67 staining) and reduced differentiation (filaggrin and cornulin staining). Active viral replication was evidenced in 3D-EpCs carrying the virus (HPV-3D-EpCs), first by the detection of the viral genome from the basal to the cornified layers (**Figure 1B**, HPV DNA) and by the detection of the replication-associated E4 and L1 viral proteins (**Figure 1B**, E4 and L1) in the upper layers, as expected. Accordingly, E6 oncoprotein expression was also detected in HPV-3D-EpCs (**Figure 1C**) as well as that of p16^INK4a^ (**Figure 1B**, lower right panel), used as a surrogate marker for the functional inactivation of pRb by the E7 oncoprotein (Keating et al., 2001). Taken together, these data show that the HPV-3D-EpCs support the viral replicative cycle, as previously reported (Meuris et al., 2016).

We next assessed the impact of HPV replication on the transcriptional expression of epithelial Cx isoforms (Di et al., 2001; Martin et al., 2014) in the epithelial sheets of 3D-EpCs. We could observe that Cx43, Cx26, Cx30, and Cx31.1 mRNA levels were significantly reduced upon HPV replication, while that of Cx30.3 remained unchanged (**Figure 1D**). In contrast, Cx45 transcripts were increased in HPV-3D-EpCs compared to control 3D-EpCs. Of note, *GJA5*, the transcript encoding the Cx40 isoform was not detected, according to its reported expression selectively in cardiac and endothelial cells (Oyamada et al., 2013). As for the Cx31 isoform, albeit *GJB3* transcripts were detected in 3D-EpCs samples, the quantitative PCR repeatedly did not pass the quality control checks, which precludes a conclusion regarding their variations in our experimental setting. Thus, while HPV replication is associated with the transcriptional downregulation of the most expressed Cx isoforms in stratified epithelia, we identify Cx45 as the only isoform whose expression is increased.

### 3.2 HPV replication induces remodeling of the subcellular and tissue Cx protein expression pattern while preserving GJ plaques

**Figure 2:**
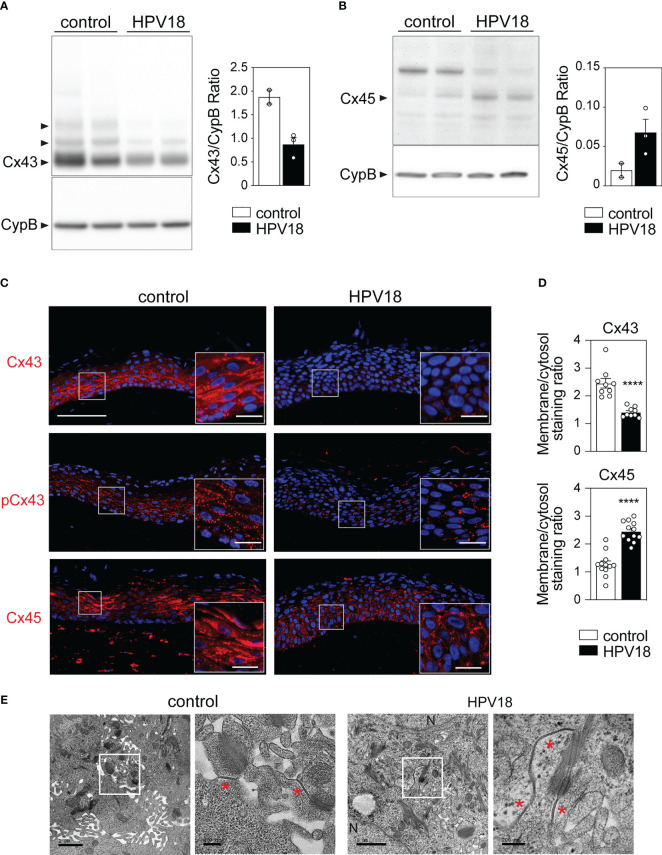
HPV-replication associated changes in Cx43 and Cx45 protein expression pattern preserves GJ plaque formation in 3D-EpCs **(A, B)** Representative Western blot analysis and densitometric quantification of Cx43 **(A)** and Cx45 **(B)** protein expression in epidermal sheets from control and HPV-3D-EpCs. Cyclophilin B (CypB) was used as loading control. Data in the densitometric analysis represent mean ± s.e.m. Data are representative of 2 independent experiments, including 2-3 3D-EpCs per group. **(C)** Representative images of control- and HPV-3D-EpCs stained for Cx43, S373-phosphorylated Cx43 (pCx43) and Cx45. Hoechst was used for nuclei staining. Insets, magnification of the selected area (white square). Scale bar=100 µm, inset scale bar=20 µm. Data are representative of 4-6 independent experiments, with n=8-16 control- and n=6-13 HPV-3D-EpCs. **(D)** Bar graphs representing the ratios of membrane over cytosolic staining for Cx43 and Cx45 in 3D-EpCs and HPV-3D-EpCs. Data represent mean ± s.e.m. obtained from the analysis of 3-4 fields of 3D-EpCs and HPV-3D-EpCs from 3 independent experiments. ****p < 0.001. **(E)** Representative electron microscopy images of control- and HPV-3D-EpC ultra-thin sections. Selected areas (white squares) are enlarged (right panels) to show gap junction plaques (red asterisks). N=Nucleus. Scale bars=1 µm and 200 nm (left and right).

We next sought to determine whether and how the transcriptional reprogramming of the Cx-encoding genes translated into quantitative modifications at the protein level. To this end, we focused on Cx43 and Cx45, the two most transcriptionnally down- and up-regulated Cx isoforms in the context of HPV replication. Western blot analyses revealed high levels of Cx43 protein in control 3D-EpCs, which were markedly reduced in HPV-3D-EpCs (**Figure 2A**). Conversely, the Cx45 isoform, barely detected in control 3D-EpCs, was increased in the context of HPV replication (**Figure 2B**) demonstrating that Cx43 and Cx45 protein levels follow the trend of their transcripts. We next performed immunofluorescence analyses to assess the potential impact of HPV replication on the tissue, cellular and subcellular distribution of Cx43 and Cx45 proteins. In control 3D-EpCs, Cx43 was mainly detected in cells from the basal and spinous layers, both in the cytoplasm and at the keratinocyte membrane (**Figure 2C**, top left panel). This pattern reproduces the one previously reported in several works (King et al., 2000; Di et al., 2001; Steinhoff et al., 2006; Brockmeyer et al., 2014; Martin et al., 2014; Garcia-Vega et al., 2021). The analysis of the phosphorylated (on Ser373) form of Cx43 known to contribute to GJ plaque growth and assembly (Dukic et al., 2018) revealed a punctuated pattern decorating the keratinocyte membrane in these tissues (**Figure 2C**, middle left panel). In HPV-replicating tissues, total Cx43 protein levels were strongly reduced in basal and spinous layers (**Figure 2C**, top right panel), and the remaining signal showed a shift from the membrane to the cytosol (**Figure 2D**, top bar graph). However, small amounts of the phosphorylated Cx43 form were still detected in basal and lower spinous layers (**Figure 2C**, middle right panel). While Cx45 protein was almost exclusively detected in keratinocyte cytoplasm and mostly restricted to the spinous and granular layers of control 3D-EpCs, which is consistent with previous reports (Garcia-Vega et al., 2021), this Cx isoform was widely redistributed in all layers of HPV-3D-EpCs with a punctuated membrane staining (**Figure 2C**, lower panels). Quantification of the membrane versus cytosolic staining confirmed the relocation of Cx45 at keratinocyte membrane in HPV-replicating tissues (**Figure 2D**, bottom bar graph). Then, we sought to determine how these changes in Cx expression and localization translated into GJ plaque presence. To this end, the suprabasal layers of control- and HPV-3D-EpCs, where Cx45 and phosphorylated Cx43 were detected, were analyzed by electron microscopy. While GJ plaques were detected in the control 3D-EpCs layers (**Figure 2E**, left panels), similar structures were also present in the HPV-3D-EpCs (**Figure 2E**, right panels), supporting the conclusion that the remodeling of Cx isoform expression in HPV-replicative epithelia allows for GJ plaque assembly.

Taken together, these results show that HPV replication is associated with a marked reorganization of Cx43 and Cx45 protein expression in stratified squamous epithelia, which preserves GJ plaques.

### 3.3 GJIC is accelerated in HPV-replicating epithelia

**Figure 3:**
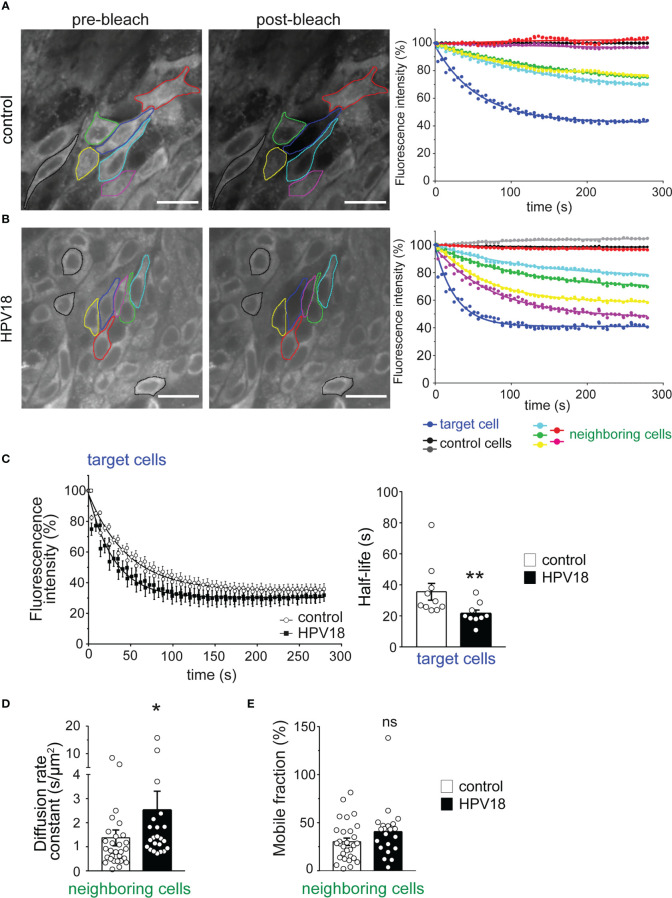
Gap-FLIP assay reveals fast cell-cell communication in HPV-3D-EpCs **(A, B)**. Epidermal sheets from control- **(A)** and HPV- **(B)** 3D-EpCs were subjected to gap-FLIP experiments. Representative images show fluorescence intensity before (pre-bleach) and after (post-bleach) laser exposure of the target cell (blue line). Direct and indirect neighboring cells (green, yellow, cyan, pink and red lines) and more distant non-connected control cells (black lines) were identified. Graphs on the right show the temporal evolution of the fluorescence as a percentage of the initial fluorescence, adjusted to a one-phase decay curve. Scale bar=20 µm. Control n=10, HPV18 n=9; 2 independent experiments. **(C)** Left, fluorescence loss of target cells over time as a one-phase decay curve in control and HPV epidermal sheets. Right, half-life values of the fluorescence in the target cells. Data represent mean ± s.e.m.; control n=10, HPV18 n=9; 2 independent experiments. **(D)** Bar graphs represent the diffusion rate constant values of the fluorescent dye in the neighboring cells. Data represent mean ± s.e.m.; control n=29, HPV18 n=22; 2 independent experiments. **(E)** Bar graphs represent the mobile fraction of the fluorescent dye in the neighboring cells. Data represent mean ± s.e.m.; control n=29, HPV18 n=22; 2 independent experiments. *p < 0.05, **p < 0.01; ns, non-significant.

We next sought to determine the impact of the HPV-induced changes in Cx expression pattern on GJIC. To quantify the dynamics of GJIC, we implemented in living 3D-EpCs the gap-FLIP assay, which allows measuring the diffusion of GJ-permeable fluorescent dye in real-time and had only been validated in monolayer cultures so far (Dukic et al., 2018). This method is based on the illumination of the targeted cell by fast repetitive flashes on a specific area, which causes the complete extinction of the intracellular dye over the time, and allows to record simultaneously the fluorescence loss in the targeted cell and neighboring cells. Epithelial sheets from control 3D-EpCs were therefore loaded with calcein dye, and one plane of the suprabasal layer was imaged in real-time upon exposure to laser bleaching. The laser was set up to hit a defined position in the cytoplasm of one identified target cell in the plane (**Figure 3A**, left panel, blue outline) and calcein dye transfer from either direct and indirect neighboring cells (**Figure 3A**, left panel, green, yellow, cyan, red and pink outlines) to the bleached target cell was assessed in control 3D-EpCs. A non-neighboring and non-communicating cell was identified in the plane to serve as internal control and correct for signal autobleaching (**Figure 3A**, left panel, black outline). Fluorescence loss of the target cell upon laser bleaching was displayed as a one-phase decay curve along the acquisition time (**Figure 3A**, right panel, blue curve) and reached a final value of 55% as compared to the control cell (**Figure 3A**, right panel, black curve). This incomplete bleaching of the target cell suggested dynamic replenishment of the dye. Accordingly, gradual loss of fluorescence was also detected in most of the neighboring cells (**Figure 3A**, right panel, green, yellow, and cyan curves), which displayed about 25 to 30% of final loss. Remarkably, certain direct (**Figure 3A**, red outline) or indirect (**Figure 3A**, pink outline) neighboring cells in the plane were non-communicating. Similar results were obtained from 10 gap-FLIP experiments performed on 3D-EpCs from 2 independent cultures. Taken together, these data support the conclusion that cells from the suprabasal layers of control 3D-EpCs display efficient GJIC.

We next applied the same methodology for characterizing calcein dye transfer between cells in HPV-3D-EpCs. This analysis revealed an overall similar profile of fluorescence loss as in control 3D-EpCs, in both target and neighboring cells (**Figure 3B**), in support of operational GJIC. The fluorescence in the target cell reached a final reduction of 60%, while that of some direct neighboring cells (**Figure 3B**, right panel, pink and yellow curves) was reduced by 40 to 50%. The analysis of additional indirect neighboring cells (**Figure 3B**, right panel, green and cyan curves) revealed that these cells also lost a significant proportion of their fluorescence (20 to 30%), as in control tissue. Finally, certain direct neighboring cells were not communicating (**Figure 3B**, red outline and curve). Similar results were obtained from 9 gap-FLIP experiments performed on HPV-3D-EpCs from 2 independent cultures. Thus, these data support the conclusion that GJIC operates efficiently in cells from HPV-replicating 3D-EpCs, albeit in a more heterogeneous manner than in control tissues. This heterogeneity might result from differential viral protein expression and/or antiviral responses at the level of the infected tissue.

Next, we performed an in-depth quantitative analysis of different parameters, including kinetic ones, characterizing GJIC. Compiling the results obtained in independent cultures revealed an accelerated loss of fluorescence in target cells from HPV-3D-EpCs as compared to their controls, as demonstrated by the slopes of the decay curves (**Figure 3C**, left panel). This observation was corroborated by significantly reduced fluorescence half-life values in target cells from HPV-3D-EpCs (**Figure 3C**, right panel). However, given the smaller size of cells in HPV-3D-EpCs (see **Figure 3B**), we further calculated the diffusion rate constant that represents the time needed for losing half of the fluorescence, a parameter that takes cell area into account. The diffusion rate constant values were significantly increased in neighboring cells from HPV-3D-EpCs (**Figure 3D**), confirming a faster cell-cell dye transfer as compared to control 3D-EpCs. The mobile fraction, which represents the percentage of fluorescence lost by neighboring cells at the end of the acquisition time, was similar between control and HPV-3D-EpCs (**Figure 3E**), demonstrating that the final level of exchange was equivalent in both types of tissue. These results collectively show that the reprogramming of the Cx expression pattern in HPV-replicating epithelia supports efficient epithelial GJIC with an intrinsic acceleration of cell exchanges.

### 3.4 HPV-associated Cx reprogramming is not achieved in 2D-culture

While the complete HPV replicative cycle requires keratinocyte differentiation and stratification, expression of HPV viral proteins can be achieved in monolayer keratinocyte cultures upon calcium-induced cell differentiation (Moody et al., 2007). We, therefore, used this approach to investigate whether the sole expression of HPV18 proteins was sufficient to promote the reprogramming of Cx43 and Cx45 expression pattern, independently of the full HPV replication upon epithelial stratification.

**Figure 4:**
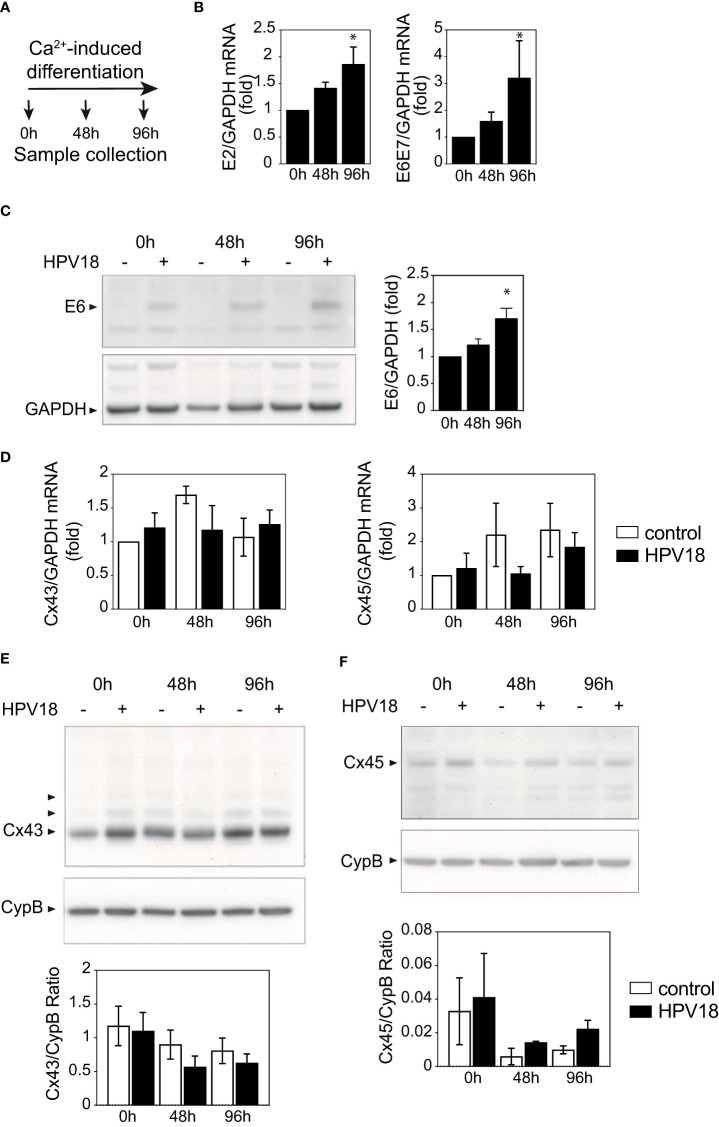
HPV protein expression is not sufficient to promote Cx expression reprogramming **(A)** Schematic representation of the Ca^2+^-induced keratinocyte differentiation protocol. **(B)** Bar graph showing the relative mRNA expression of *E2* and *E6E7* viral genes in Ca-Diff keratinocytes over the differentiation time. Data represents mean ± s.e.m.; 3-4 independent experiments. *p < 0.05. **(C)** Representative Western blot of viral E6 protein expression and densitometric analysis of Cx43 protein expression in Ca^2+^-induced differentiated keratinocyte monolayer cell cultures over the differentiation time. GAPDH was used as loading control. Representative image of one experiment out of 3. Data in the densitometric analysis represent mean ± s.e.m.; control n=3, HPV18 n=3. **(D)** Bar graphs showing the relative mRNA expression of *Cx43/GJA1* and *Cx45/GJC1* genes in Ca-Diff keratinocytes over the differentiation time. Data represents mean ± s.e.m.; 4 independent experiments. **(E, F)** Representative Western blots of Cx43 **(E)** and Cx45 **(F)** expression in Ca^2+^-induced differentiated keratinocyte monolayer cell cultures over the differentiation time. Cyclophilin B (CypB) was used as a loading control. Bar graphs show the densitometric analyses **(E, F)**, that correspond to the median +/- interquartile range from 2 independent experiments.

The non-differentiated NIKS keratinocytes transfected or not with circularized HPV18 genome that were used to generate 3D-EpCs (**Figure 1A**) displayed similar levels of Cx43 and Cx45 mRNA (**Figure S2A**) and substantial expression of Cx43 protein was detected in both Western blot (**Figure S2B**) and immunofluorescence staining (**Figure S2C**). These cells were exposed to a calcium-containing medium, without feeder fibroblasts, and harvested after 48h and 96h of culture (**Figure 4A**). The efficiency of the calcium-induced keratinocyte differentiation process was confirmed by the increased expression of the early *E2* viral gene and *E6E7* oncogene transcripts (**Figure 4B**) that was validated at the protein level for the E6 protein (**Figure 4C**). However, no changes in the transcript levels of the Cx43 and Cx45 mRNA transcript levels were observed (**Figure 4D**). At the protein level, Cx43 expression was also stable over calcium-induced keratinocyte differentiation (**Figure 4E**), while Cx45 protein levels were impacted by differentiation and tended to be higher in HPV18-expressing keratinocytes as compared to their controls after 96h (**Figure 4F**), supporting the hypothesis of post-transcriptional Cx45 regulation.

Thus, the expression of HPV18 proteins *per se* was insufficient to recapitulate Cx transcriptional and protein reprogramming in differentiated keratinocytes, suggesting that this viral-dependent process occurs only in stratified tissues, upon completion of the HPV18 replicative cycle.

### 3.5 Cx expression and distribution are reprogrammed in HPV-positive biopsies

**Figure 5:**
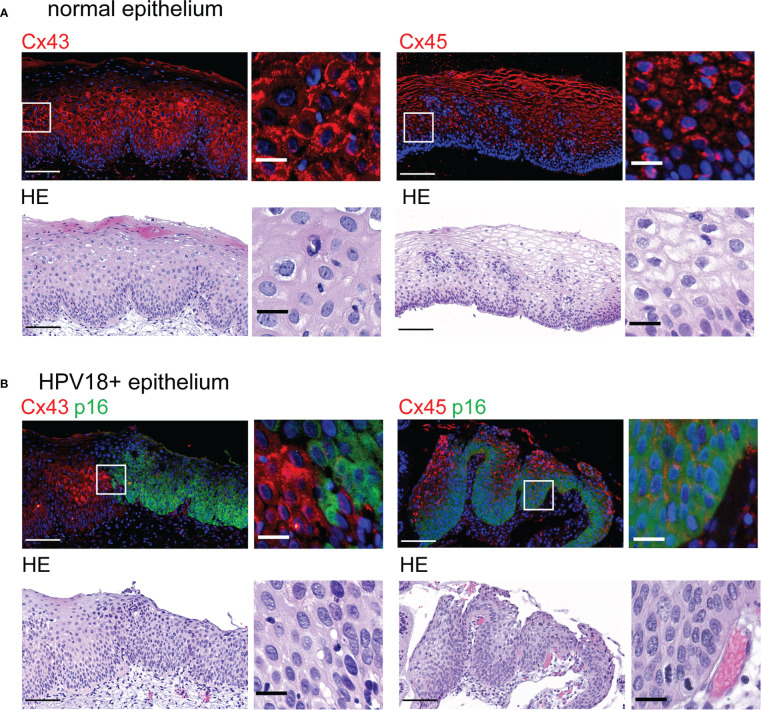
Redistribution of Cx43 and Cx45 protein expression in HPV-infected cervical tissues **(A)**. Representative images of normal cervical stratified epithelium stained with Cx43- and Cx45-specific antibodies (top left and right, respectively) and hematoxylin-eosin (HE) (bottom). Representative images of normal cervical stratified epithelium stained for Cx43 (top left) and Cx45 (top right). Insets show enlargement of Cx43 and Cx45 staining in the selected areas (white squares). Scale bars=100 µm and 20 µm for large tissue views and insets, respectively. Data are representative of 3-4 independent biopsies. **(B)** Representative images of HPV18-positive CIN1 biopsies stained with p16^INK4a^ (p16)-specific antibodies and with either Cx43- or Cx45-specific antibodies (top left and right, respectively). Hoechst was used for nuclei staining. HE staining is shown (bottom panels). Enlarged images from selected regions (white squares) are shown on the right. Scale bars=100 µm and 20 µm for large tissue views and insets. Data are representative of 2-3 independent biopsies.

We finally sought to extend our analyses to HPV18-positive cervical biopsies. We focused on HPV18-positive pre-cancerous CIN1 (**Figure 5**) that are reported to still replicate the virus (Lowy and Schiller, 2006; Schiffman et al., 2016).

As in other stratified epithelia (Di et al., 2001), control squamous cervical tissue displayed Cx43 expression mainly in the spinous layer as punctuated staining detected at the cell membrane (**Figure 5A**, top left). Occasionally, Cx43 expression was also cytoplasmic or in cells of the basal layer. As for Cx45 staining, it was almost exclusively found in the spinous and granular layers of cervical squamous epithelia and mostly localized in keratinocyte cytoplasm (**Figure 5A**, top right).

In CIN1 biopsies, we first delineated the HPV-positive areas according to p16^INK4a^ expression (Schiffman et al., 2016), which is associated with HPV18 replication in HPV-3D-EpCs (**Figure 1B**). In p16^INK4a^-positive regions, Cx43 expression was barely detected in contrast to adjacent p16^INK4a^-negative areas (**Figure 5B**, top left panels). Immunofluorescence analyses also revealed changes in cellular and subcellular localization of Cx45 between p16^INK4a^-positive and -negative regions. Indeed, Cx45 expression was redistributed to the keratinocyte membrane in HPV-positive lesion areas, whereas it remained mostly cytoplasmic in the upper layers of the control epithelium (**Figure 5B**, top right panel). Thus, a reorganization of Cx43 and Cx45 expression and distribution is detected in areas of human cervical tissues that express HPV. These results support the physiological relevance of the findings obtained in control- and HPV-3D-EpCs.

## 4 Discussion

In this work, we aimed to investigate the consequences of HPV replication on GJIC in stratified squamous epithelia. Therefore, we have set up real-time quantification of GJIC dynamics in living 3D-EpCs, the sole culture model supporting the full replicative cycle of HPV, by adapting a non-invasive and very sensitive imaging method based on FLIP. This technical breakthrough enabled us to demonstrate efficient GJIC in the context of HPV replication in living stratified epithelia and the resulting broad reprogramming of the Cx expression and distribution pattern.

We have provided several lines of evidence supporting the suitability of 3D-EpCs as tissue models to reproduce the pattern of Cx expression and distribution in the human epithelium (King et al., 2000; Di et al., 2001; Steinhoff et al., 2006; Brockmeyer et al., 2014; Martin et al., 2014; Garcia-Vega et al., 2021). In these living 3D models of stratified epithelia, we pioneered the analysis of GJIC dynamics by adapting an imaging method based on FLIP. In our setting, the whole tissue is loaded with calcein dye, the laser is set up to hit one target cell in one plane in the suprabasal layer of the tissue, and imaging of the neighboring cells is performed in the same plane. Thus, this allows for visualizing GJIC between cells in defined planes of the 3D-EpCs, considered as representative regions of these tissues. We have chosen to image the suprabasal region of the 3D-EpCs, as the best compromise between technical constraints, due to limitations of measurement by the laser depth, and scientific interest, as cells in the suprabasal layer are particularly impacted in terms of Cx43 and Cx45 levels and distribution and are known to express most early viral proteins during HPV replication. Using this sensitive and live approach, we were able to quantify dye exchanges between cells in this layer of 3D-EpCs and to evidence efficient GJIC, whether they were, or not, replicating HPV18. While these results were somehow unexpected considering the marked decrease in the RNA levels of most epithelial Cx isoforms, as well as the strong downregulation of the most abundant one, Cx43, they are supported by the detection of GJ plaques along the suprabasal layers of both control- and HPV-3D-EpCs by electron microscopy. A previous study reported decreased expression of Cx43, Cx30, and Cx26 proteins in HPV-associated dysplastic cervical tissues (Aasen et al., 2005), and an association between the loss of Cx43 expression and GJIC, assessed by a semiquantitative method (King et al., 2000), was proposed. Both works referred to alterations resulting from HPV carcinogenesis, a rare event in the life cycle of those highly prevalent commensal viruses. Here, we question for the first time the impact of HPV replication on GJIC efficiency, by directly comparing epithelia that only differ in terms of viral replication.

Refined analysis of GJIC dynamics demonstrated qualitative changes, with an intrinsic acceleration of this process between communicating cells from HPV-3D-EpCs as compared to their controls. These changes are most likely due to the differential contribution of Cx isoforms in connexons from HPV- versus control-3D-EpCs, resulting from Cx transcriptional reprogramming as well as Cx protein subcellular and tissue distribution. In HPV-replicating tissues, the Cx45 protein was upregulated and redistributed to all epithelial layers, with a shift of its cellular localization toward the cell membrane suggesting its participation in GJ plaques. Together with the reduction of Cx43 protein levels, these results are consistent with a contribution of Cx45 to GJIC in the suprabasal layers of HPV-3D-EpCs. The low amounts of Cx43 remaining in HPV-3D-EpCs suggest its reduced involvement in GJIC. However, the detection in basal and lower spinous layers of small amounts of a phosphorylated Cx43 form associated with GJ plaque assembly (Solan and Lampe, 2014; Dukic et al., 2018) indicates that this Cx isoform could still contribute to GJIC in HPV-3D-EpCs. Indeed, Cx gene expression is controlled at the transcriptional and post-transcriptional levels, and Cx protein isoforms are further submitted to post-translational modifications that control their turnover, trafficking, and, ultimately, function (Aasen et al., 2018; Laird and Lampe, 2018). One possibility would be that Cx45 and Cx43 form heteromeric and heterotypic connexons with specific properties (Koval et al., 2014), as reported in cardiac tissue (Desplantez, 2017). Such a process might account for the intrinsic acceleration of GJIC in cells from HPV-3D-EpCs compared to their controls. While the existence of such connexons is supported by the coexistence of Cx45 and Cx43 at the keratinocyte membrane in the basal and suprabasal layers of HPV-3D-EpCs, where most keratinocytes are expressing HPV proteins, their contribution to GJIC remains to be directly assessed. Also, the potential changes in charge and size selectivity of these connexons, and as a consequence in the nature of the exchanged molecules, require investigation. In addition, other Cx isoforms may also contribute to efficient GJIC in control - and HPV-3D-EpCs. The complete characterization of Cx protein isoforms expression and distribution in HPV-3D-EpCs will deserve further investigation.

The transcriptional reprogramming of *GJA1* (encoding for Cx43) and *GJC1* (encoding for Cx45) could not be achieved in calcium-differentiated 2D cultures of keratinocytes, despite HPV protein expression, supporting the conclusion that this expression is not sufficient to induce Cx-encoding gene transcriptional modulation in keratinocytes. Our results are consistent with RNASeq analyses of HPV16-expressing keratinocytes differentiated in the presence of calcium, which indicated no alteration of *GJA1* and *GJC1* transcript levels, in contrast to *GJB2* (encoding for Cx26)*, GJB5* (encoding for Cx31.1) and *GJB6* (encoding for Cx30) transcripts that were reduced (Klymenko et al., 2017). This supports the conclusion that transcriptional *GJA1* down- and *GJC1* up-regulation critically depend on epithelium stratification and genuine HPV replication as achieved in 3D-EpCs, the sole culture system allowing for a complete HPV life cycle, including viral particle production (Lambert et al., 2005; Meuris et al., 2016). The mechanisms involved may include differential activation of transcription factors along epithelial differentiation and/or epigenetic regulation of Cx genes transcription (Oyamada et al., 2013), together with HPV active replication. As for Cx43 protein levels, they were not reduced in HPV18-expressing calcium-differentiated keratinocytes, as opposed to what was reported in cervical tumor cell lines expressing the HPV18 and HPV16 E6 oncoprotein (Sun et al., 2015). In the latter work, HPV16 E6 affected Cx43 expression levels and trafficking to the cell membrane by binding to a complex formed by a human homolog of *Drosophila* Discs Large protein and Cx43, a process that might have been fostered by E6 overexpression in transfected or transformed cell lines. In HPV18-expressing calcium-differentiated keratinocytes, Cx45 protein levels tended to be higher than in their controls, suggesting that HPV expression may be a driver of Cx45 protein increase in keratinocytes. Importantly, regarding subcellular and tissue distribution, the expression patterns of Cx43 and Cx45 were similar in HPV-3D-EpCs as in HPV18-positive CIN1 biopsies, with low levels of Cx43 expression and membrane localization but increased detection of Cx45 at the cell membrane. Altogether, these results emphasize the relevance of 3D-EpCs for performing functional assays aimed at studying the Cx-associated pathophysiological consequences of HPV replication.

While HPV-3D-EpCs do not recapitulate the complexity of the HPV virome in human epithelia, they reproduce HPV productive infection found in low-grade intraepithelial lesions (*i.e.* CIN1 (Schiffman et al., 2016)), underlining their relevance in modeling the interplay between human stratified squamous epithelia and the replicative life cycle of HPV. In this context, our results show coordinated mechanisms promoting GJIC in HPV-replicating epithelia, that may contribute to the control of the virus life cycle in the context of mild dysplasia. Whether Cx modulation at the transcriptional, post-transcriptional, and post-translational levels during HPV replication is virus-driven or reflects infected cell response to infection remains to be explored, as well as the pathophysiological relevance of such modulation. Cx43 and Cx45 have been shown to spread the cyclic GAMP(2’-5’) second messenger between keratinocytes during viral infection (Ablasser et al., 2013), and Cx43 can transfer immunogenic peptides from keratinocytes to Langerhans cells (Neijssen et al., 2005). Thus, increased Cx45 trafficking at the cell membrane may be viewed as a compensatory mechanism to support GJIC despite the reduction in Cx43 and could be an epithelial defense mechanism favoring immune defense to contain HPV infections into a commensal state. In the lungs, GJIC has also been shown to spread suppressive signals between epithelial cells and alveolar macrophages (Westphalen et al., 2014), suggesting that GJIC could also play a role in limiting inflammation toward the epithelial virome, and possibly other members of the microbiota. HPVs have co-evolved with their human hosts for thousands of years. The extent to which the replication of different HPV types colonizing healthy epithelia may imprint tissue and immune cells, thereby contributing to epithelial homeostasis, immune training, and protective host defense mechanisms, remains an open avenue to explore (Chen et al., 2018; Strickley et al., 2019; Koster et al., 2022; Lebeau et al., 2022). Given the high turnover dynamics of Cxs (Laird and Lampe, 2018) and the emerging complexity of viral and other microbial communities that colonize epithelia in health and disease (Tirosh et al., 2018; Kabashima et al., 2019), it will be important to characterize the outcome of HPV replication in terms of Cx expression, isoform combinations, and associated functions. Indeed, in the present study, we have focused on GJIC as the functional consequence of Cx reprogramming, but the formation of hemichannels communicating with the extracellular environment (Aasen et al., 2017; Laird and Lampe, 2018) and/or Cx interactome (Fumagalli et al., 2020) may also be important outcomes of Cx remodeling in HPV-replicating epithelia.

In conclusion, our work unravels the functional adaptation of stratified squamous epithelia to HPV replication and sheds light on the coordinated Cx reprogramming that supports efficient GJIC, a process that might be important in anti-viral responses controlling epithelial virome, and more broadly in shaping epithelial responses towards microbiota.

## Data availability statement

The original contributions presented in the study are included in the article/**Supplementary Material**. Further inquiries can be directed to the corresponding authors.

## Ethics statement

The studies involving human participants were reviewed and approved by CHU-ULiege. Written informed consent for participation was not required for this study in accordance with the national legislation and the institutional requirements.

## Author contributions

CG, FB, and GS-L contributed to the conception and design of the study. CG, AJ-R, ML, FM-N, SD, AF, MH, PR, GP, FB, and GS-L contributed to the investigation and formal analysis of the results. CG, FB, and GS-L wrote the first draft of the manuscript. All authors contributed to the article and approved the submitted version.

## Supplementary material

Supplementary Figure 1 HPV replication modifies keratinocyte proliferation and differentiation in 3D-EpCs Representative images of control and HPV-3D-EpCs stained for HE (top), Ki-67 (middle) as a proliferation marker, and filaggrin and cornulin (bottom) as differentiation markers in the granular layer. Scale bar =100 µm. Control n = 10-13, HPV18 n = 7-10; 3-4 independent experiments.

Supplementary Figure 2 Undifferentiated keratinocytes carrying the HPV18 genome do not display altered levels of Cx43 and Cx45. **(A)** Bar graphs show the relative mRNA expression of Cx43/GJA1 and Cx45/GJC1 genes in Ca-Diff keratinocytes over the differentiation time. Data represents mean±s.e.m.; control n=4, HPV18 n=4. **(B)** Representative Western blot and densitometric analysis of Cx43 protein expression in undifferentiated keratinocyte monolayer cell cultures. Data in the densitometric analysis represent mean±s.e.m.; control n = 4, HPV18 n = 4. **(C)** Representative images of undifferentiated keratinocyte monolayer cell cultures stained for Cx43. Scale bar = 50 µm.
