# Supplementary material for: Reprogramming of connexin landscape fosters fast gap junction intercellular communication in human papillomavirus-infected epithelia
Source: Front Cell Infect Microbiol. 2023 May 16;13:1138232. doi: 10.3389/fcimb.2023.1138232 (PMC10228504; doi:10.3389/fcimb.2023.1138232)
Supplement: Supplementary file 1 [file Table_1.docx]

Supplementary Material

Reprogramming of connexin landscape fosters fast gap junction intercellular communication in human papillomavirus-infected epithelia

Carmen Gallego, Agnieszka Jaracz-Ros, Marta Laganà, Françoise Mercier-Nomé, Séverine Domenichini, Amos Fumagalli, Philippe Roingeard, Michael Herfs, Guillaume Pidoux, Françoise Bachelerie*, Géraldine Schlecht-Louf*

*** Correspondence:**

Françoise Bachelerie

francoise.bachelerie@universite-paris-saclay.fr

Géraldine Schlecht-Louf

[geraldine.schlecht-louf@[universite-paris-saclay.fr](mailto:geraldine.schlecht-louf@universite-paris-saclay.fr)](mailto:geraldine.schlecht-louf@u-psud.fr)

**Supplementary Table 1. List of primers, probes, and assay IDs**

| **Name, sequence** | **Catalog Number, Company** |
| --- | --- |
| E6E7 Forward  5’ CAC-AAT-GTT-GTG-TAT-GTG-TTG-TAA-GTG 3’ | NA, Eurogentec |
| E6E7 Reverse  5’ GGT-CGT-CTG-CTG-AGC-TTT-CTA 3’ | NA, Eurogentec |
| UPL #131 for E6E7 | Cat#04694155001, Roche |
| E2 Forward  5’ GGG-AAC-ATG-GCA-TAC-AGA-CA 3’ | NA, Eurogentec |
| E2 Reverse  5’ GCC-ATT-TGC-AGT-TCA-ATA-GCT-T 3’ | NA, Eurogentec |
| UPL #129 for E2 | Cat#04693655001, Roche |
| GAPDH Forward  5’ GCA-CAA-GAG-GAA-GAG-AGA-GAC-C 3’ | NA, Eurogentec |
| GAPDH Reverse  5’ AGG-GGA-GAT-TCA-GTG-TGG-TG 3’ | NA, Eurogentec |
| UPL #3 for GAPDH | Cat#04685008001, Roche |
| TaqMan™Gene Expression Assay: Cx43/GJA1 | Assay ID: Hs00748445_s1, Applied biosystems |
| TaqMan™Gene Expression Assay: Cx26/GJB2 | Assay ID: Hs00955889_m1, Applied biosystems |
| TaqMan™Gene Expression Assay: Cx30.3/GJB4 | Assay ID: Hs00920816_s1, Applied biosystems |
| TaqMan™Gene Expression Assay: Cx31.1/GJB5 | Assay ID: Hs00193376_m1, Applied biosystems |
| TaqMan™Gene Expression Assay: Cx30/GJB6 | Assay ID: Hs00917676_m1, Applied biosystems |
| TaqMan™Gene Expression Assay: Cx45/GJC1 | Assay ID: Hs00271416_s1, Applied biosystems |
| TaqMan™Gene Expression Assay: GAPDH | Assay ID; Hs02758991_g1, Applied biosystems |
