# Supplementary material for: Reprogramming of connexin landscape fosters fast gap junction intercellular communication in human papillomavirus-infected epithelia
Source: Front Cell Infect Microbiol. 2023 May 16;13:1138232. doi: 10.3389/fcimb.2023.1138232 (PMC10228504; doi:10.3389/fcimb.2023.1138232)
Supplement: Supplementary file 2 [file Table_2.docx]

Supplementary Material

Reprogramming of connexin landscape fosters fast gap junction intercellular communication in human papillomavirus-infected epithelia

Carmen Gallego, Agnieszka Jaracz-Ros, Marta Laganà, Françoise Mercier-Nomé, Séverine Domenichini, Amos Fumagalli, Philippe Roingeard, Michael Herfs, Guillaume Pidoux, Françoise Bachelerie*, Géraldine Schlecht-Louf*

*** Correspondence:**

Françoise Bachelerie

francoise.bachelerie@universite-paris-saclay.fr

Géraldine Schlecht-Louf

[geraldine.schlecht-louf@[universite-paris-saclay.fr](mailto:geraldine.schlecht-louf@universite-paris-saclay.fr)](mailto:geraldine.schlecht-louf@u-psud.fr)

**Supplementary Table 2. List of reagents and resources**

| REAGENT or RESOURCE | SOURCE | IDENTIFIER |
| --- | --- | --- |
| **Antibodies** | | |
| Mouse monoclonal anti-Tubulin (clone DM1A) | Sigma-Aldrich | Cat#T6199; RRID:AB_477583 |
| Mouse monoclonal anti-GAPDH (clone FF26A) | Thermo Fisher Scientific | Cat# 14-9523-82; RRID:AB_11151331 |
| Mouse monoclonal anti-Cyclophilin B (clone CL3901) | Thermo Fisher Scientific | Cat# MA5-31392; RRID:AB_2787028 |
| Rabbit polyclonal anti-Connexin-43 | Sigma-Aldrich | Cat#C6219; RRID:AB_476857 |
| Rabbit polyclonal anti-phospho-Connexin 43 (Ser373) | Invitrogen | Cat#PA5-64670; RRID:AB_2662693 |
| Rabbit polyclonal anti-Connexin-45/GJC1 | Novus, R&D | Cat#NBP2-76925 |
| Mouse monoclonal Connexin-45/GJC1 (clone G-7) | Santa Cruz Biotechnology | Cat# sc-374354 ; RRID:AB_10988777 |
| Rabbit monoclonal anti-Ki67 (clone SP6) | Abcam | Cat#ab16667; RRID:AB_302459 |
| Mouse monoclonal anti-Filaggrin (clone FLG/1561) | Abcam | Cat#ab218395 |
| Mouse monoclonal anti-p16 INK4A (clone JC8) | Santa Cruz Biotechnology | Cat#sc-56330   RRID:AB 785018 |
| Rabbit polyclonal anti- cornulin (CRNN) | Proteintech | Cat#11799-1-AP |
| Rabbit polyclonal anti-HPV18-E4 | J. Doorbar | N/A |
| Mouse anti-HPV18-E6 (clone AVC#402) | Arbor Vita Corporation | N/A |
| Mouse Monoclonal Anti- HPV (clone K1H8) | Dako Agilent | Cat#M3528 |
| Amersham ECL Mouse IgG, HRP-linked F(ab')₂ fragment (from sheep) | GE Healthcare | Cat#NA9310; RRID:AB_772193 |
| Goat anti-Mouse IgG, Alexa Fluor 594 | Invitrogen | Cat#A-11005; RRID:AB 141372 |
| Amersham ECL Rabbit IgG, HRP-linked F(ab')₂ fragment (from donkey) | GE Healthcare | Cat#NA9340; RRID:AB772191 |
| Goat anti-Rabbit IgG, Alexa Fluor 594 | Invitrogen | Cat#A-11012; RRID:AB 141359 |
| Goat anti-Mouse IgG (H+L) Highly Cross-Adsorbed Secondary Antibody, Alexa Fluor™ 488 | Invitrogen | Cat#A-11029  RRID:AB 2534088 |
| **Biological Samples** |  |  |
| Human cervical tissue specimens, female | Biobank (University Hospital Centre of Liege, Belgium) | N/A |
| **Chemicals, Peptides, and Recombinant Proteins** | | |
| Blasticidin | Invivogen | Cat#ant-bl-1 |
| Hydrocortisone | Calbiochem | Cat#386698; CAS:50-23-7 |
| Adenine hydrochloride | Sigma-Aldrich | Cat#A9795; CAS:2922-28-3 |
| Insulin | Sigma-Aldrich | Cat#I6634; CAS:11070-73-8 |
| Cholera toxin | Sigma-Aldrich | Cat#C8052; CAS:9012-63-9 |
| EGF | Sigma-Aldrich | Cat#E9644; CAS:62253-63-8 |
| Collagen | Merck | Cat#08-115 |
| 1,2-dioctanoyl-sn-glycerol (C8:0) | Calbiochem | Cat#317505; CAS:60514-48-9 |
| Calcein Red-Orange AM | Invitrogen | Cat#C34851; CAS: [148504-34-1](https://www.sigmaaldrich.com/FR/fr/search/148504-34-1?focus=products&page=1&perpage=30&sort=relevance&term=148504-34-1&type=cas_number) |
| **Experimental Models: Cell Lines** | | |
| Human: NIKS (nearly-diploid immortalized keratinocytes) | P.F. Lambert | N/A |
| Mouse: 3T3-J2 fibroblasts | P.F. Lambert | N/A |
| Human: NHDF (normal human dermal fibroblasts) from neonatal foreskin | Lonza | Cat#CC-2509 |
| **Oligonucleotides** | | |
| Cx43 Forward  5’ TCT-GAG-TGC-CTG-AAC-TTG-CC 3’ | This paper, Eurogentec | N/A |
| Cx43 Reverse  5’ CCC-TCC-GCA-GTT-GAG-TAG-G 3’ | This paper, Eurogentec | N/A |
| GAPDH Forward  5’ GGG-AAA-CTG-TGG-CGT-GAT 3’ | This paper, Eurogentec | N/A |
| GAPDH Reverse  5’ GGA-GCA-GTG-GGT-GTC-GCT-GTT 3’ | This paper, Eurogentec | N/A |
| Primers, probes and Taqman gene assays, see Supplementary Table 1 | This paper | N/A |
| **Recombinant DNA** | | |
| pBS-HPV18 | J. Doorbar |  |
| pCDNA6 Blasti | J. Doorbar |  |
| **Software and Algorithms** | | |
| Graphpad Prism (version 7) | GraphPad Software | www.graphpad.com |
| Fiji | (58, 59) | https://fiji.sc/ |
| NDP.scan and NDP.view2 | Hamamatsu |  |
| Motion 2D | Mobyle web portal | http://mobyle-serpico.rennes.inria.fr/ |
