# Supplementary figures and images for: Reprogramming of connexin landscape fosters fast gap junction intercellular communication in human papillomavirus-infected epithelia
Source: Front Cell Infect Microbiol. 2023 May 16;13:1138232. doi: 10.3389/fcimb.2023.1138232 (PMC10228504; doi:10.3389/fcimb.2023.1138232)

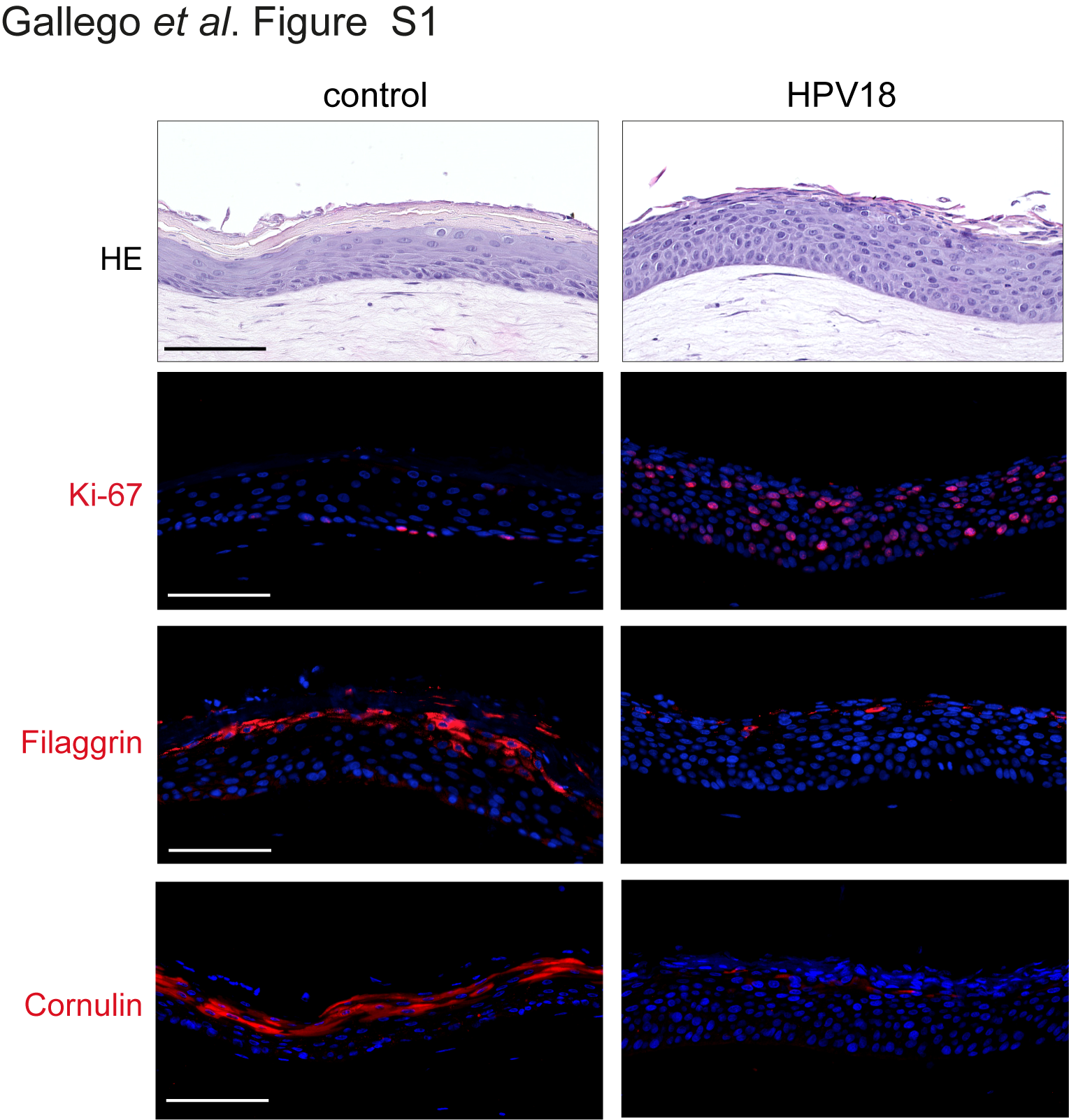

Supplement: Supplementary Figure 1 [file Image_1.tif]

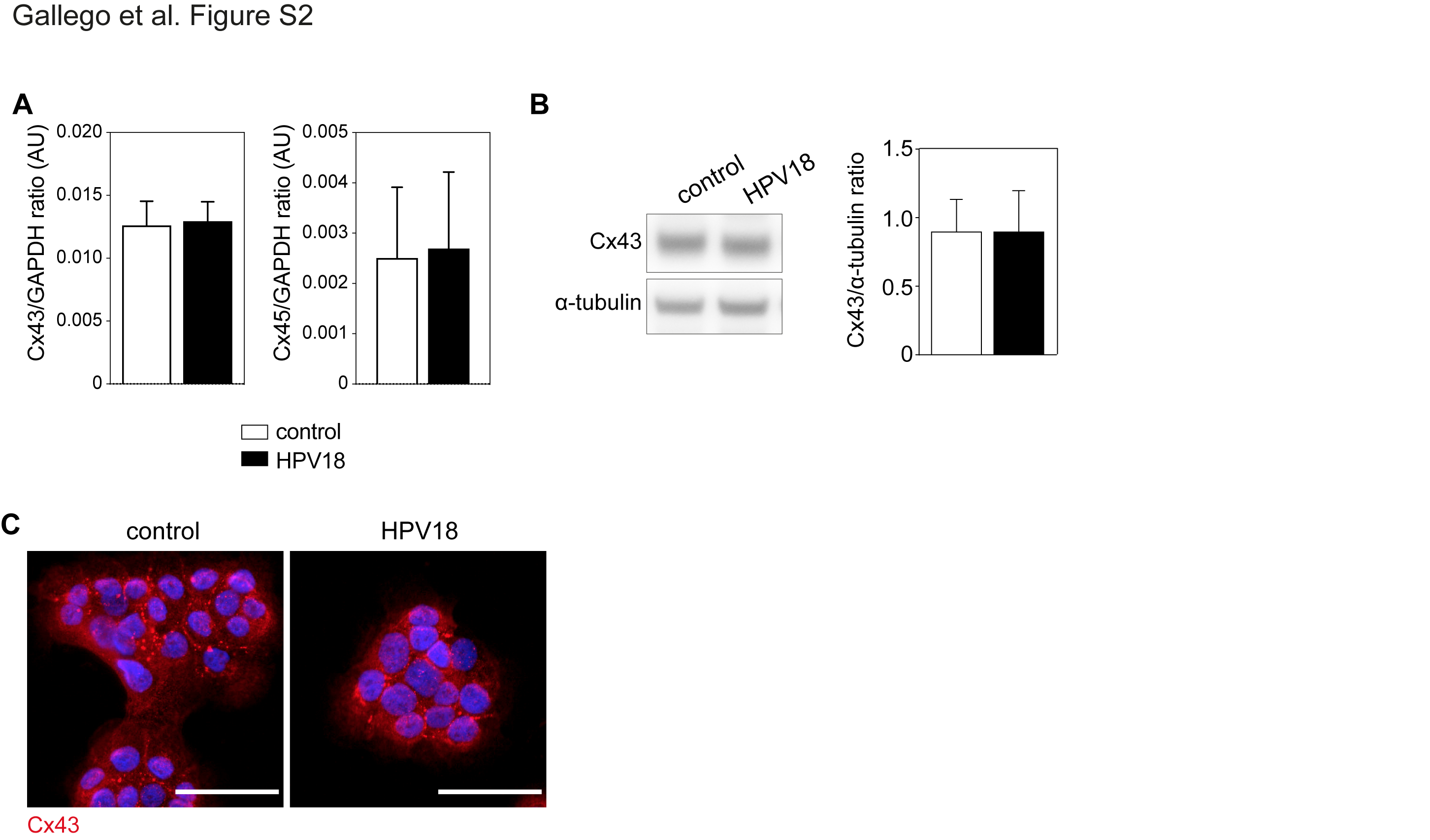

Supplement: Supplementary Figure 2 [file Image_2.tif]
